# Effectiveness of Topical Hemocoagulase on the Healing of Surgical Wounds in the Neck: A Randomized Clinical Trial

**DOI:** 10.1007/s12663-025-02623-z

**Published:** 2025-07-19

**Authors:** Dyna Albert, M. R. Muthusekhar

**Affiliations:** Department of Oral and Maxillofacial Surgery, Saveetha Institute of Medical and Technical Sciences, 162, Poonamallee High Rd, Velappanchavadi, Chennai, Tamil Nadu 600077 India

**Keywords:** Hemocoagulase, DermaLab Combo, Neck incision, Head and neck cancer, Oral cancer, Wound healing

## Abstract

**Background:**

This study aims to evaluate the role and effectiveness of topical hemocoagulase in the healing process of surgical neck wounds in oral cancer patients undergoing surgery as the primary treatment modality.

**Methodology:**

This prospective, parallel, double-blinded randomized clinical trial was conducted on oral cancer patients planned for surgical resection and neck dissection. The study included 22 patients divided into two groups: Group I received topical hemocoagulase, and Group II served as the control. Quantitative assessment was performed using the DermaLab Combo device to measure dermal thickness, collagen level, elasticity, viscoelasticity, and retraction time. Qualitative assessment was performed using the Patient and Observer Scar Assessment Scale (POSAS).

**Results:**

Analysis of quantitative variables revealed a significant improvement in dermal thickness, collagen levels, elasticity, viscoelasticity, and retraction time in Group I compared to Group II at both the 1st and 4th postoperative weeks. Qualitative assessment using POSAS showed a notable reduction in pain and improved overall scar appearance in the hemocoagulase group. These findings are consistent with previous studies that have shown the effectiveness of hemocoagulase in enhancing wound healing and reducing scar formation.

**Conclusion:**

Topical hemocoagulase significantly enhances wound healing in surgical neck wounds of oral cancer patients, providing both quantitative improvements and higher patient satisfaction.

## Introduction

The healing of surgical wounds is a complex and multifaceted process that is of particular importance in the management of oral cancer patients undergoing neck dissection and resection. In recent years, there has been a growing interest in the use of topical agents to enhance wound healing, with hemocoagulase emerging as a potential therapeutic option due to its dual hemostatic and wound healing properties [1].

Hemocoagulase has traditionally been used for its hemostatic properties in various surgical procedures. Its application in wound healing is supported by its ability to promote clot formation and enhance collagen synthesis, both of which are essential for the reparative processes of wound healing [2]. Despite these promising attributes, there is limited clinical evidence regarding its efficacy in head and neck surgeries, particularly in patients with oral cancer. To address this gap, robust clinical assessment methods are required.

**Fig. 1:**
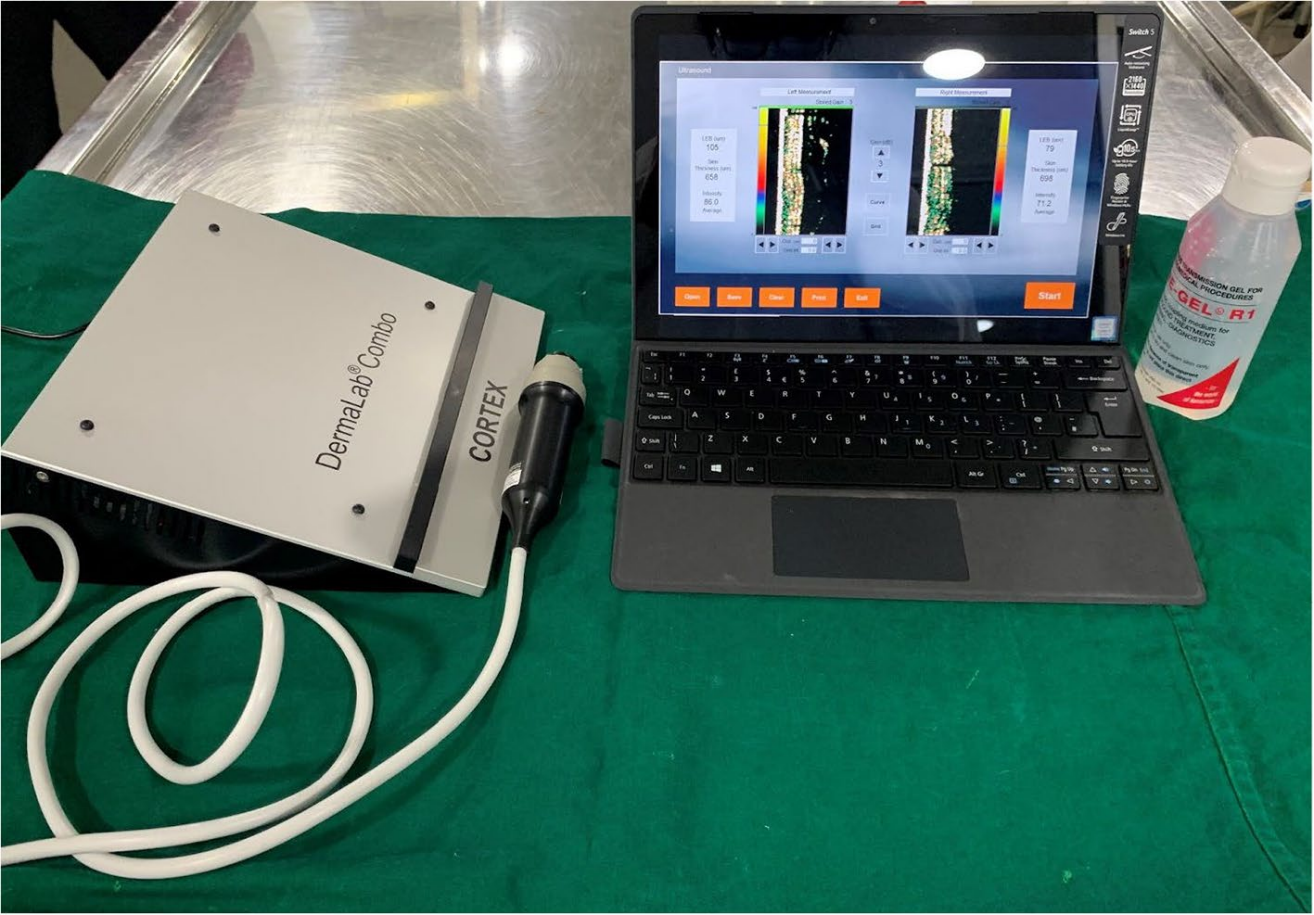
DermaLab Combo Unit

The assessment of scar formation and wound healing typically involves both qualitative and quantitative measures. Quantitative tools such as the DermaLab Combo (DLC) (Fig. 1) provide objective data on parameters like dermal thickness, collagen levels, and skin elasticity, which are invaluable for clinical research. On the other hand, qualitative tools like the Patient and Observer Scar Assessment Scale (POSAS) capture the subjective experiences and satisfaction of patients, which are crucial for comprehensive patient care [3–6].

This study employs a randomized clinical trial design to evaluate the effectiveness of topical hemocoagulase in enhancing primary wound healing in the neck region of oral cancer patients following neck dissection. Specifically, it aims to: (1) quantitatively measure differences in scar and adjacent normal tissue in terms of dermal thickness, collagen level, and mechanical properties (elasticity, viscoelasticity, and retraction time) using ultrasound and elasticity probes of DLC. (2) qualitatively evaluate the patient and observer satisfaction of the wound healing using the patient observer scar assessment scale (POSAS). The uniqueness of this study lies in its comprehensive approach, combining both non-invasive quantitative assessment and subjective qualitative evaluation, specifically focused on primary wound healing.

## Materials and Methods

### Study Design and Population

This prospective, parallel, double-blinded randomized clinical trial was conducted on patients diagnosed with oral cancer and planned for surgical resection along with neck dissection. Patients who reported to the Department of Oral Oncology, were included after obtaining informed consent.

### Ethical Clearance

The study was approved by the Scientific Review Board and Institutional Human Ethical Committee (SRB/SDC/OSURG-1902/20/TH-01 and IHEC/SDC/OMFS-1902/20/413). The trial was registered in the Clinical Trial Registry of India prior to commencement (CTRI/2020/11/029267).

### Sample Size Calculation

Sample size was calculated using G-power software, based on the post hoc analysis of mean and standard deviation from a study by Thamboo et al. (2009), with a power set at 90% and an alpha error of 0.05. The sample size was determined to be 22, with 11 individuals in each group  [6].

### Method of Patient Selection

Patients reporting to the Department of Oral Oncology and diagnosed with oral cancer were included based on inclusion and exclusion criteria. Patients were enrolled and randomized into two groups by the primary investigator: Gr-I (topical hemocoagulase) and Gr-II (control) using computer generated sequence for randomization along with the Sequentially Numbered, Opaque, Sealed Envelope (SNOSE) method for allocation concealment.

### Criteria for Patient Selection

#### Inclusion Criteria

- Diagnosed with oral cancer in stages I, II, or III according to the AJCC eighth edition.
- Scheduled for neck dissection along with surgical resection.
- Both genders, aged 30–70 years, with no known allergies to topical hemocoagulase.
- Able to comprehend the Patient Observer Scar Assessment Scale (POSAS) independently or with assistance.

#### Exclusion Criteria

- Recurrent oral cancer or new primary malignant tumors of the head and neck.
- Occult metastasis to the neck after surgical resection of a primary malignancy.
- Bilateral neck dissection, simultaneous microvascular reconstruction, or microvascular reconstruction for pre-existing defects.
- Benign tumors, dermatological diseases, cardiac pacemakers, bleeding disorders, coagulopathy, vascular anomalies, myocardial infarction, or undergoing anticoagulant therapy.
- Uncontrolled diabetes and/or systemic hypertension at the time of surgery.

### Outcome Measures

#### Primary Outcome

The primary outcomes of this study were aimed to quantitatively assess the difference in dermal thickness, collagen level, Young's modulus of elasticity, viscoelasticity and retraction time of the surgical scar in the neck and adjacent normal tissue in patients of both groups using the ultrasound probe (USGP) and elasticity probe (EP) of DLC at 1st and 4th postoperative week.

#### Secondary Outcome

The secondary outcome of this study was to qualitatively assess the patient and observer perspective of the surgical scar in the neck using POSAS on 1st and 4th postoperative week.

### Procedure

Demographic data with respect to age, gender, site of primary tumor, histopathological description, clinical TNM staging, type of neck dissection and incision employed were collected. The patients included in the study were blinded and randomly divided into 2 groups. The study group Gr-I included patients for whom 2 ml of topical hemocoagulase (Botroclot®, Juggat Pharma) was applied at the site of the neck incision after completion of subcutaneous layer suturing. Each milliliter of Botroclot contains 0.2 CU of hemocoagulase, along with chlorhexidine gluconate solution IP 0.1% v/v as a preservative, and water for injection IP q.s. The solution was allowed to soak for 8–10 minutes before proceeding to skin layer closure. The control group Gr-II included patients for whom the closure of the neck incision proceeded without any intervention. In both the groups, to avoid bias, 3–0 vicryl was used for subcutaneous layer closure and 3–0 polypropylene was used for skin layer closure. Similarly, in both groups, simple buried suturing technique was used for subcutaneous layer closure and vertical mattress suturing technique was used for skin layer closure. All the patients had an uneventful postoperative recovery and were shifted to the Intensive Care Unit immediately after the surgery where they were monitored for 1–2 days before being shifted to the ward. Postoperative antibiotic therapy consisted of intravenous infusion of 1.5 g Magnus Forte and 80 mg Gentamicin, twice daily along with 500 mg Metronidazole, thrice daily. All the patients remained as in-patients for a minimum of 7 days after the surgery and conventional wound dressings with normal saline, betadine solution, betadine ointment and gauze were given every day. On discharge from the hospital, patients were instructed to maintain clean the surgical site and topical application of betadine ointment over the surgical site in the neck was advised once daily for both groups. The sutures were removed alternatively on postoperative day 7 and day 10.

**Fig. 2:**
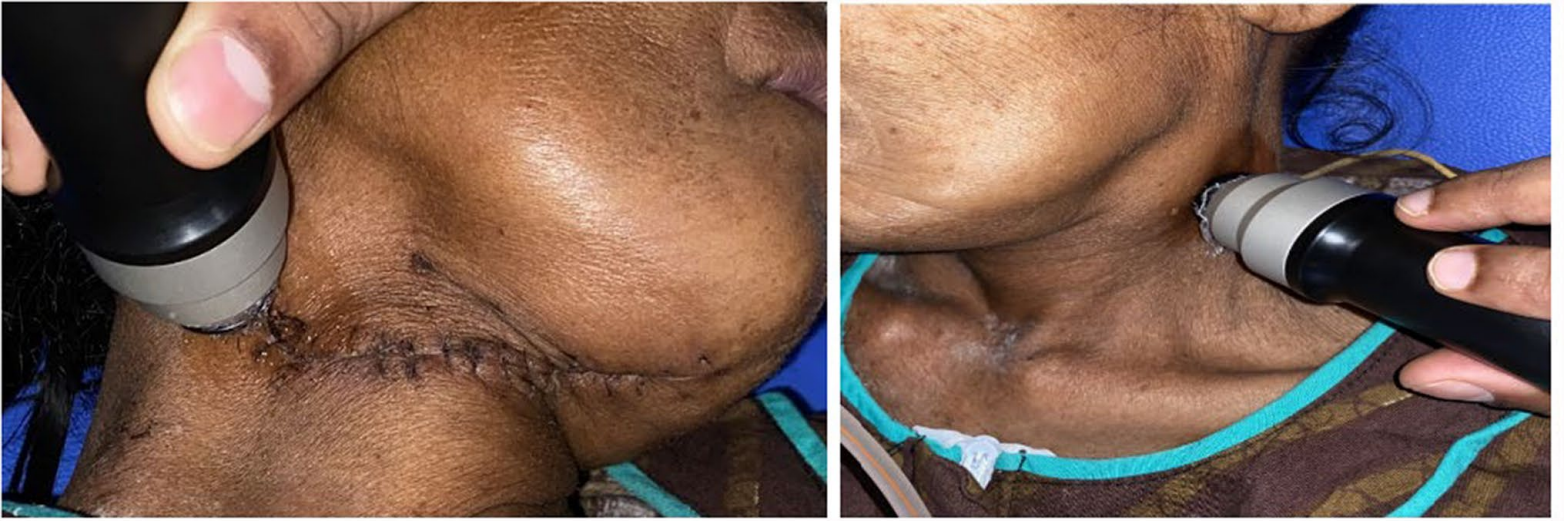
Quantitative assessment of scar characteristics using ultrasound probe of DermaLab Combo at scar and contralateral normal site

**Fig. 3:**
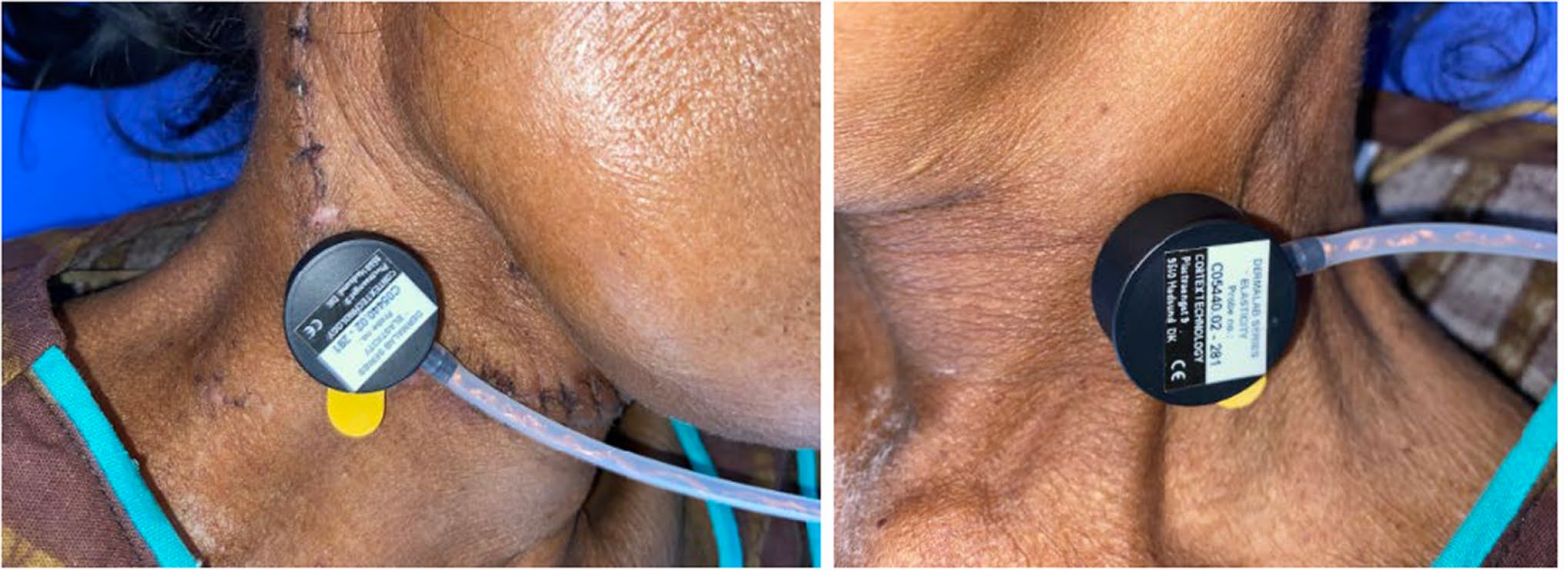
Quantitative assessment of scar characteristics using elasticity probe of DermaLab Combo at scar site and contralateral normal site

The USGP measured dermal thickness and collagen levels, while EP assessed Young's modulus, viscoelasticity, and retraction time of the scar. Each measurement was taken three times on both scar and contralateral normal tissue for accuracy (Figs. 2 and 3). The jelly-coated USGP was held perpendicular to the skin, with thickness displayed on the monitor. EP, with a 10 mm suction aperture, was set to 'normal skin' mode at 3 mbar pressure for standardization. Each measurement involved three elevation/retraction cycles, with average values used for analysis. While the elasticity measurements were automatically averaged, dermal thickness and collagen levels required manual calculation.

**Fig. 4:**
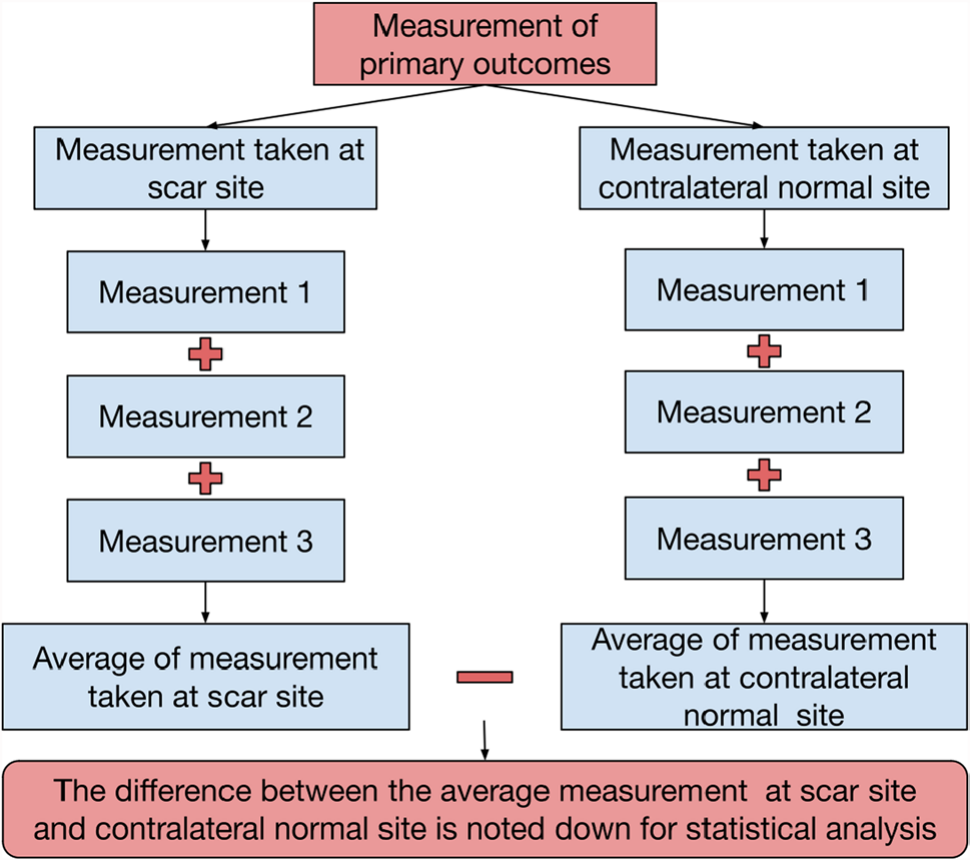
Flowchart depicting the methodology

Measurements were taken at two time points by a blinded assessor: 1 week and 4 weeks postoperatively which allowed for assessment of both early and later stages of scar healing. For each patient, every parameter in the primary outcome was measured at the site of neck incision and contralateral normal neck skin after which the difference between them was calculated thereby providing a quantitative measure of how the healing scar differed from normal skin. By using each patient's own contralateral normal site as a reference, this method effectively controls for individual variability, thereby reducing potential bias and the impact of confounding factors. A smaller difference between the scar and normal skin was considered indicative of a better outcome, whereas a larger difference was viewed as a poorer outcome (Fig. 4). At the same visit, POSAS was recorded by the blinded patient and the blinded assessor. Thus the precise, quantitative measurements from the DLC device were combined with the qualitative assessments from the POSAS.

### Statistical Analysis

The data were entered in Microsoft Excel Spreadsheet and analyzed using IBM SPSS (Version 20.0, Armonk, NY, USA: IBM SPSS for Windows). Normality of data was assessed using Kolmogorov–Smirnov test. The demographic data was analyzed using descriptive statistics while mean and standard deviation were used to summarize the quantitative data. Frequency and percentage were used to summarize qualitative and categorical data. Independent samples t-test was used for intergroup comparison of the primary outcomes  while Mann–Whitney U test was used for intergroup comparison of the POSAS scores. Effect sizes were quantified using Cohen's d, and 95% confidence intervals were calculated to assess the precision of the estimated effects. Throughout the study, a p value of < 0.05 was considered as a statistically significant difference.

## Results

Enrolment, group allocation, and follow-up of participants extended between 2021 and 2023. Based on the selection criteria, the study included 22 patients (17 male, 5 female) with a mean age of 48.7 years and nil attrition. Buccal mucosa (40.9%) was the most common primary tumor site. Stage II disease was most prevalent (45.5%), and selective neck dissection was performed in 59.1% of cases (Table 1). Primary outcomes showed statistically significant improvement (*p* = 0.001) in the topical hemocoagulase group (Gr-I) compared to the control group (Gr-II) on postoperative days (POD) 7 and 28 for Young's modulus of elasticity, viscoelasticity, tissue retraction time, and collagen level intensity. Dermal thickness showed no statistical significance between groups (Table 2).

**Table 1:** Demographic characteristics of the participants

Mean age	48.7 years (range: 30–70 years)
Gender distribution	Male: 17 (77.3%), Female: 5 (22.7%)
Male to female ratio	3.4:1
Primary tumour site	Buccal mucosa (40.9%) Lateral border of tongue (36.4%) Gingivobuccal sulcus (13.6%) Retromolar trigone (9.1%)
Histopathology	Squamous cell carcinoma (100%)
Pathological grade	Well differentiated (63.6%) Moderately differentiated (18.2%) Poorly differentiated (13.6%) Superficially invasive (4.5%)
TNM Staging (AJCC 8th Edition)	Stage I (13.6%) Stage II (45.5%) Stage III (40.9%)
Type of neck dissection	Selective neck dissection (59.1%) Modified radical neck dissection (40.9%)
Type of incision used	Modified Schobinger (13.6%) Neck Crease (13.6%) Modified Schobinger + Lip Split (4.5%) Utility (63.6%) Utility + Lip Split (4.5%)

**Table 2:** Comparison of quantitative and qualitative variables between two groups over time

	Parameter	Group I Day 7	Group II Day 7	Day 7	Group I Day 28	Group II Day 28	Day 28
	Quantitative Variables	Mean ± SD	Mean ± SD	P value	Mean ± SD	Mean ± SD	*P* value
1	Young's Modulus (MPa)	1.76 ± 0.34	4.43 ± 0.87	0.001*	0.60 ± 0.26	2.57 ± 0.74	0.001*
2	Viscoelasticity (MPa)	2.25 ± 0.49	6.08 ± 1.02	0.001*	0.42 ± 0.09	2.55 ± 0.43	0.001*
3	Collagen Level	39.89 ± 6.05	65.48 ± 9.95	0.001*	30.35 ± 9.18	60.65 ± 9.61	0.001*
4	Retraction Time (ms)	31.18 ± 12.06	71.0 ± 29.01	0.001*	14.91 ± 2.84	33.55 ± 8.88	0.001*
5	Dermal Thickness (microns)	575.0 ± 316.28	819.14 ± 396.61	0.16	314.66 ± 245.98	601.05 ± 385.63	0.15

	Qualitative Variables	Mean (Mean Rank)	Mean (Mean Rank)	*P* value	Mean (Mean Rank)	Mean (Mean Rank)	*P* value
*POSAS: Patient Satisfaction*							
1	Pain	5.10 (6.95)	6.64 (16.05)	0.001*	3.10 (10.68)	3.36 (12.32)	0.56
2	Itching	5.91 (10.36)	6.36 (12.64)	0.43	3.10 (10.95)	3.27 (12.05)	0.69
3	Color Difference	6.82 (9.73)	7.18 (14.09)	0.21	5.73 (13.68)	4.82 (9.32)	0.11
4	Stiffness	6.27 (8.91)	7.18 (14.09)	0.06	4.82 (10.00)	5.18 (13.00)	0.30
5	Thickness	5.91 (8.45)	7.27 (14.55)	0.06	4.45 (9.32)	5.00 (13.68)	0.11
6	Irregularity	6.09 (8.09)	7.45 (14.91)	0.01*	4.55 (9.91)	5.10 (13.10)	0.27
7	Overall Opinion	6.09 (7.18)	7.55 (15.82)	0.001*	4.55 (10.41)	5.10 (12.59)	0.43
*POSAS: Observer Satisfaction*							
1	Vascularity	6.09 (7.18)	7.55 (15.82)	0.08	4.55 (10.41)	4.91 (12.59)	0.30
2	Pigmentation	6.64 (9.00)	7.27 (14.00)	0.56	4.91 (10.00)	5.45 (13.00)	0.79
3	Thickness	7.00 (12.32)	6.82 (10.68)	0.15	5.18 (11.09)	5.36 (11.91)	0.09
3	Relief	6.45 (9.45)	7.18 (13.55)	0.07	4.55 (9.14)	5.55 (13.86)	0.06
4	Pliability	6.27 (9.05)	7.09 (13.95)	0.004*	4.55 (8.86)	5.36 (14.14)	0.06
5	Surface Area	6.09 (7.64)	7.36 (15.36)	0.016*	4.64 (8.86)	5.55 (14.14)	0.13
6	Overall Opinion	6.18 (8.18)	7.18 (14.82)	0.002*	5.00 (9.41)	5.55 (13.59)	0.21

For secondary outcomes, at POD 7, the observer scores for pliability (*p* = 0.004), surface area (*p* = 0.016) and overall opinion (*p* = 0.002) were significantly better in Gr-I. Patient scores for pain (*p* = 0.001), irregularity (*p* = 0.01) and overall opinion (*p* = 0.001) also favoured Gr-I. By POD 28, no observer or patient POSAS domains differed significantly (Table 2).

Effect size estimates for primary outcomes indicated that at Days 7 and 28, Gr-I exhibited large reductions in Young’s modulus (*d* = − 4.04, − 3.55) and viscoelasticity (*d* = − 4.79, − 6.86). Collagen levels (*d* = − 3.11, − 3.22) and retraction time (*d* = − 1.79, − 2.83) were also significantly closer to normal skin than in Gr-II. However, dermal thickness changes were minimal (*d* = − 0.68, − 0.89; 95% CIs overlapping zero) (Table 3).

**Table 3:** Effect sizes and confidence intervals for quantitative variables across days 7 and 28

Parameter	Day 7			Day 28		
	Cohen's d	CI Lower	CI Upper	Cohen's d	CI Lower	CI Upper
Young's Modulus (MPa)	− 4.04	− 3.28	− 2.06	− 3.55	− 2.48	− 1.46
Viscoelasticity (MPa)	− 4.79	− 4.56	− 3.10	− 6.86	− 2.42	− 1.84
Collagen Level	− 3.11	− 33.01	− 18.17	− 3.22	− 38.66	− 21.94
Retraction Time (ms)	− 1.79	− 60.23	− 19.41	− 2.83	− 24.76	− 12.52
Dermal Thickness (microns)	− 0.68	− 564.21	75.93	− 0.89	− 577.38	4.60

## Discussion

Head and neck oncological surgeries often require lengthy neck incisions, which can lead to significant scarring and functional restrictions due to the neck's high mobility [7]. The healing process is further complicated in cancer patients due to their compromised health status and potential previous treatments. While various scar reduction methods exist, their applicability to recently treated head and neck cancer patients is questionable. Topical hemocoagulase, a thrombin-like enzyme, has emerged as a promising agent for wound healing. Its affordability, availability, ease of use, biocompatibility, and dual benefit of hemostasis and wound healing make it suitable for use in head and neck oncological surgeries [8]. Albert et al. (2022) conducted a systematic review that suggested a positive role of topical hemocoagulase in improving wound healing [9].

Hemocoagulase, derived from snake venom, acts through a dual mechanism involving thrombin-like activity and Factor X activation, promoting clot formation and rapid hemostasis [10]. Furthermore, it enhances collagen synthesis, crucial for wound healing and improved scar quality, making it beneficial in surgical wounds, particularly in head and neck oncological surgeries [11]. In this study, topical application of hemocoagulase significantly improved dermal thickness, collagen levels, elasticity, viscoelasticity, and retraction time in surgical neck wounds of oral cancer patients, as confirmed by quantitative assessments using the DermaLab Combo. Qualitative assessments also demonstrated notable pain reduction and improved overall scar appearance, aligning with the findings of Thamboo et al. [6].

While hemocoagulase offers therapeutic benefits, potential side effects such as hypersensitivity reactions, local irritation, and systemic coagulopathy must be considered. Drug interactions, especially with anticoagulants, are a concern due to hemocoagulase's pro-coagulative properties. Therefore, careful patient selection and monitoring are essential. This randomized clinical trial provides evidence for the effectiveness of topical hemocoagulase in enhancing wound healing in surgical neck wounds, bringing out its potential role in improving patient satisfaction and quantitative outcomes while minimizing risks.

This study's results align with previous research demonstrating the positive effects of topical hemocoagulase on wound healing [12–14]. However, our approach is unique in its comprehensive assessment of both qualitative and quantitative aspects of wound healing. We utilized the POSAS for qualitative evaluation, addressing both patient and observer perspectives. For quantitative assessment, we employed the DLC device, which provided non-invasive measurements of dermal thickness, collagen level, elasticity, and tissue retraction time, adhering to ethical guidelines for human research [15–17]. Interestingly, patients treated with topical hemocoagulase reported significantly reduced pain on postoperative day 7, consistent with findings from Aslam et al. This observation warrants further investigation into the potential analgesic properties of topical hemocoagulase. Our study is novel in evaluating the role of topical hemocoagulase in primary wound healing, as opposed to previous human trials that assessed healing by secondary intention [12–14]. The intraoperative application of topical hemocoagulase before skin closure minimizes discomfort for the patient and doesn't require special maintenance.

Quantitative results showed that topical hemocoagulase significantly improved collagen level and elasticity (measured as Young's modulus, viscoelasticity, and tissue retraction time) on both postoperative days 7 and 28. These parameters demonstrated increasing differences over time, as indicated by larger effect sizes and narrower confidence intervals, suggesting a progressively stronger and more consistent impact of the intervention. Specifically, the reductions in Young’s modulus and viscoelasticity indicate markedly improved scar pliability while collagen and retraction time values approaching those of normal skin suggest enhanced remodelling toward physiological tissue characteristics when topical hemocoagulase was used. However, dermal thickness exhibited smaller effect sizes and wider confidence intervals, indicating greater variability. This contrast highlights the consistent and growing influence of the intervention on mechanical properties and structural integrity, except for dermal thickness, which displayed distinct behaviour. Qualitative assessment revealed significant reductions in pain, surface irregularity and overall opinion of the neck wound on postoperative day 7.

Our study was limited by sample size and follow-up duration, which was restricted to four weeks to avoid the confounding factor of potential chemoradiation treatment. Nonetheless, we effectively mitigated the influence of the relatively small sample size and the unbalanced distribution of gender and age by using within-subject comparison whereby scar characteristics were compared against the contralateral normal skin and the difference between them was analysed. Even though the follow-up period was focused on the early stages of healing, our robust methodology allowed us to capture meaningful differences in scar outcomes during this critical period which are likely indicative of long-term scar quality, providing valuable insights into the effectiveness of topical hemocoagulase in improving wound healing in neck incisions.

## Conclusion

In conclusion, topical hemocoagulase significantly improved both quantitative and qualitative characteristics of neck wounds in head and neck oncological surgeries. We found it to enhance collagen levels, elasticity, and reduce pain and surface irregularity in the early postoperative period. These findings suggest that topical hemocoagulase is effective in promoting the healing of surgical wounds in the neck among patients undergoing head and neck cancer surgeries. Future studies in various clinical settings could further expand our understanding and application of this agent in wound healing.
